# Ellagic Acid Modulates Necroptosis, Autophagy, Inflammations, and Stress to Ameliorate Nonalcoholic Liver Fatty Disease in a Rat Model

**DOI:** 10.1002/fsn3.4694

**Published:** 2025-01-19

**Authors:** Zhuoheng Li, Juan Li, Shuli He, Jun Chen, Chengjun Deng, Jintao Duan

**Affiliations:** ^1^ Gastroenterology Department Kunming Children's Hospital Kunming China

**Keywords:** autophagy, ellagic acid, metabolism, NAFLD, necroptosis

## Abstract

Nonalcoholic fatty liver disease (NAFLD) is considered one of the most common metabolic disorders worldwide. Although the pathoetiology of NAFLD is not fully elucidated, recent evidence suggests the involvement of stress, inflammation, and programmed death in the onset and progression of the disease. This investigation aimed to evaluate the effects of ellagic acid (EA), a known herbal antioxidant, on a high‐fat diet (HFD)‐induced animal model of NAFLD by evaluating the status of lipid profile, necroptosis (RIPK1, RIPK3, and MLKL), autophagy (LC3, ATG5, and BECN1), inflammation (TNF‐α, IL‐6, IL‐4, and IL‐10), and stress (SOD, CAT, GR, GPx, and MDA). In this regard, rats were randomly divided into 6 groups as follows: normal diet controls, HFD (supplemented with high caloric diet model), EA low dose (HFD and 10 mg/kg/day EA), EA middle dose (HFD and 25 mg/kg/day EA), EA high dose (HFD and 50 mg/kg/day EA), and Rosiglitazone (HFD and 10 mg/kg/day Rosi). After the treatment, the levels of markers related to necroptosis and autophagy in the liver tissue as well as the lipid profiles, inflammation, and oxidative stress status were analyzed. It was shown that the dose of EA was able to improve the weight gain and lipid profile when compared to NAFLD animals (*p*‐value < 0.001). Moreover, EA increased the level of LC3 and ATG5 while decreasing BECN 1, RIPK1, RIPK3, and MLKL compared to the HFD‐induced NAFLD rats (*p*‐value < 0.05). TNF‐α and IL‐6 were decreased after EA administration, whereas IL‐4 and IL‐10 levels were increased (*p*‐value < 0.001). Furthermore, the increase in the activity of SOD, CAT, GR, and GPx along with the decrease in MDA levels indicated the suppression of oxidative stress by EA treatment compared to the NAFLD rats (*p*‐value < 0.0001). The current findings may suggest that EA improves NAFLD via modulation of necroptosis, autophagy, inflammation, and stress.

## Graphical abstract

Nonalcoholic fatty liver disease (NAFLD) is considered one of the most common metabolic disorders worldwide. Although the pathoetiology of NAFLD is not fully elucidated, recent evidence suggests the involvement of stress, inflammation, and programmed cell death in the onset and progression of the disease. The present study aimed to evaluate the effects of ellagic acid (EA), a known herbal antioxidant, on a high‐fat diet (HFD)‐induced rat model of NAFLD by evaluating the status of lipid profile, necroptosis (RIPK1, RIPK3, and MLKL), autophagy (LC3, ATG5, and BECN1), inflammation (TNF‐α, IL‐6, IL‐4, and IL‐10), and stress (SOD, CAT, GR, GPx, and MDA).

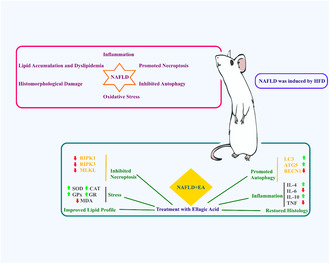

## 1 Introduction

With a global prevalence of ~25%, nonalcoholic fatty liver disease (NAFLD), a chronic liver disorder, is a serious concern for healthcare providers (Bassal et al. 2022; Younossi et al. 2016). A plethora of evidence strongly associates NAFLD with metabolic disorders such as central obesity, dyslipidemia, hypercholesterolemia, type 2 diabetes, and metabolic syndrome (Zarghamravanbakhsh, Frenkel, and Poretsky 2021). The higher prevalence of NAFLD in men in the general population compared to women, as well as recent reports related to “lean NAFLD” have made it possible that genetic factors are involved in the disease onset and progression (Zou et al. 2020). Contrary to the old hypothesis known as the “two‐hit hypothesis”, recent findings consider NAFLD as a systematic disorder that has a complex multi‐hit pathophysiology with various stages—from steatohepatitis to hepatocellular carcinoma. The complexity of the disease is mainly related to cytokines, adipokines, innate immune activation, lipotoxicity, microbiome, environment, and genetic factors (Meli, Mattace Raso, and Calignano 2014; Svegliati‐Baroni et al. 2019).

The management of NAFLD generally depends on preventing lipid accumulation, suppressing inflammation and stress, and dealing with tissue fibrosis. Moreover, regular exercise and diet control are the most important approaches to disease control. However, a definitive treatment for NAFLD has not been approved and the molecular mechanisms underlying the occurrence and progression of the disease are being investigated by ongoing studies (Rong et al. 2023; Tarantino, Citro, and Capone 2019). Comorbidities and potential adverse effects of current therapeutical options led researchers to assess the efficacy of herbs as these compounds represent desirable properties such as anti‐inflammatory and antioxidant functions (Jayanti et al. 2024).

Ellagic acid (EA), a polyphenol found in different fruits (e.g., raspberries, strawberries, grapes, and pomegranates) and nuts (e.g., walnuts, pecans, pistachios, and acorns), is a dilactone possessing both hydrophilic moiety and lipophilic moiety (Aishwarya, Solaipriya, and Sivaramakrishnan 2021; Ríos et al. 2018; Rossi et al. 1991). The specific structure of EA enables this nonflavonoid compound to accept electrons, hence contributing to antioxidant redox reactions (Rossi et al. 1991; Vakili, Rostami, and Rastegar 2024). Interestingly, recent findings demonstrate that EA has protective effects against disorders related to liver tissue. In addition, experimental, review, and meta‐analysis studies have suggested EA's function in the management of glucose and lipid metabolism, improving the expression of effector genes, and treating NAFLD. Although recent findings indicate the involvement of programmed cell death programs in the progression of chronic diseases including NAFLD (Samare‐Najaf et al. 2023a; Sun et al. 2024), the effect of EA on these mechanisms remains unclear.

Necroptosis is a type of regulated cell death program dependent on receptor‐interacting protein kinases (RIPKs). Necroptosis, with three major components including RIPK1, RIPK3, and mixed lineage kinase domain‐like protein (MLKL), has been immensely recently investigated in various liver diseases such as NAFLD (Sun et al. 2024; Xinyu et al. 2023). Moreover, autophagy is a catabolic process described as a double‐edged sword since it contributes to both the survival and death of the cells (Samare‐Najaf et al. 2023b). Recently, various studies have suggested that modification of autophagy may represent a novel approach to managing liver‐related disorders including NAFLD (An et al. 2023; Wu, Zhang, and Chan 2018).

Despite the significant prevalence of NAFLD, efforts continue to propose a novel treatment strategy with high efficiency and acceptable safety. Cell death programs as well as inflammation and stress pivotally contribute to the occurrence and progression of NAFLD, and on the other hand, EA has previously been shown to efficiently improve NAFLD in animal models. However, the effect of EA on regulated cell death mechanisms is not elucidated (Altamimi et al. 2022; Elseweidy et al. 2022). Hence, the present study aimed to establish an animal model of NAFLD and evaluate the effects of different doses of EA by measuring physical and biochemical characteristics and markers related to necroptosis, autophagy, inflammation, and oxidative stress.

## 2 Materials and Methods

### 2.1 Chemicals

Normal and high‐fat diets (HFD) were provided by Sinopharm Chemical Reagent Company, Shanghai, China. Rosiglitazone was purchased from Sigma Aldrich, xylazine was purchased from Alfasan, Woerden, Holland, and ketamine was purchased from Bremer Pharma GMBH, 34414 Warburg, Germany.

### 2.2 Animal Care and Handling

Thirty‐six healthy male *Sprague–Dawley* rats, 180–200 g, were provided by the University Animal Care and Handling Center. The provided rats were kept in polypropylene cages on a 12/12 h day and night cycle, at 22°C ± 3°C, and acclimatized with the environment and investigators for 1 week prior to the commencement of the treatment. The standard pellet diet was provided with fresh drinking water *ad libitum*.

### 2.3 Study Design and Treatments

Once the animals were in the laboratory condition, rats were randomly divided into 6 groups (*N* = 6) including control (CON, fed with normal diet and carboxymethylcellulose), HFD (fed with high caloric diet), EA low dose (EA‐L, received HFD and EA orally 10 mg/kg/day), EA middle dose (EA‐M, received HFD with EA 25 mg/kg/day), EA high dose (EA‐H, received HFD and EA 100 mg/kg/day), and Rosiglitazone (Rosi, administered HFD and Rosi, 10 mg/kg/day). The HFD treatment continued for 4 weeks to induce NAFLD, and 5 weeks of HFD treatment plus oral administration of EA and rosiglitazone was performed. EA and rosiglitazone were suspended in carboxymethylcellulose (0.5% solution). The NAFLD induction, doses, and duration of treatment are selected according to previous studies (Qin et al. 2018; Zeb and Akbar 2018).

### 2.4 Sample Collection

After 9 weeks, all animals were sacrificed under xylazine 2% and ketamine 10% anesthesia, 5 mL blood samples were collected by cardiac puncture, and serum samples were isolated after incubation at room temperature for 15 min and centrifugation at 2000 RPM. Serum samples were aliquoted and stored at −20°C for further biochemical analyses. Moreover, liver tissues from all animals were removed, washed with sterile PBS, and stored at −80°C for further analyses, and for histological study isolated liver tissues were preserved in formaldehyde.

### 2.5 Histological Examination

Liver samples were fixed and embedded in 10% formaldehyde and paraffin, respectively, and then sections with 5 μm thickness were prepared after staining with H&E (hematoxylin and eosin) and Masson'strichrome. Consequently, Axiovision, Zeiss Germany computerized image analysis system was used to assess the count according to a previous study. Steatosis was graded according to a previously published article as severe > 60%, moderate = 30%–60%, and mild = 5%–30% of liver cells affected, hence it was analyzed using a morphological semi‐quantitative method (Kleiner et al. 2005). Moreover, the liver tissues were assessed for histological characteristics such as sinusoidal fibrosis, vacuolation, acidophilic necrosis, polymorph nuclear infiltration, and ballooning degeneration (El‐Lakkany et al. 2016; Suzuki and Toledo‐Pereyra 1993).

### 2.6 The Measurement of Body Weight and Biochemical Indices

Before the sacrification of animals, final body weights were analyzed. Moreover, the key biochemical components (LDL [low‐density lipoprotein], TC [total cholesterol], HDL [high‐density lipoprotein], and TG [triglyceride]) were measured in serum and liver tissue, according to the manufacturer's guidelines.

### 2.7 RT‐qPCR Analysis

Total RNA was extracted from hepatic tissues using a specific kit purchased from Thermo Fisher, USA (the PureLink RNA Mini Kit) adhering to the manufacturer's directions. The RNA's purity and integrity were assessed with the Biotek Nanodrop system and agarose 1.5% gel electrophoresis. cDNA synthesis was performed by the High‐Capacity cDNA Reverse Transcription Kit (Thermo Fisher, USA), and then real‐time quantitative PCR (RT‐qPCR) analysis was conducted using the StepOne Real‐Time PCR System (Applied Biosystems, USA) and Maxima SYBR Green qPCR Master Mix (Thermo Fisher, USA). Expression levels were normalized relative to β‐actin. The 2^−ΔΔCT^ method was used to present obtained data as fold changes. The specific primer sequences associated with genes encoding necroptosis (*RIPK1, RIPK3, MLKL*) and autophagy (*LC3*, *ATG5*, *BECN1*) markers were provided to perform RT‐qPCR analysis (Table 1). A two‐stage reaction protocol was executed, commencing with a 95°C denaturation stage for 5 min, followed by 40 cycles of 95°C for 20 s and 60°C for 15 s.

**TABLE 1:** Primer sequences.

Primer	Sequence
β‐Actin forward	5′‐CCAATCTATGAGGGTTACGC‐3′
β‐Actin reverse	5′‐TTCAATGTCACGCACGATTC‐3′
RIPK1 forward	5′‐GAGCTGGACTGCGGTATTGAG‐3′
RIPK1 reverse	5′‐AACCATGACCCGTCCCTTGA‐3′
RIPK3 forward	5′‐AGCGTTATGGAGTCGTCTAA‐3
RIPK3 reverse	5′‐AATCTGCACTTCGGTATTCGG‐3′
MLKL forward	5′‐CATCATGAGCCGTTCATTGA‐3′
MLKL reverse	5′‐AAGTCTGACTTGAACTCTCGAC‐3
LC3 forward	5′‐CATGCCGTCCGAGAAGACCT‐3′
LC3 reverse	5′‐GATGAGCCGGACATCTTCCACT‐3′
ATG5 forward	5′‐CCTATCTGA TGTCCCTAGC‐3′
ATG5 reverse	5′‐GGTTTCTATGAGGGTTAGAGC‐3′
BECN1 forward	5′‐TGAGGAATGGAGGGGTCTAA‐3′
BECN1 reverse	5′‐TGGGCTGTGGTAAGTAATGG‐3′

### 2.8 Measurement of Necroptotic and Autophagic Markers

The current study benefited from available kits based on the enzyme‐linked immunosorbent assay (ELISA) to measure the markers related to necroptosis and autophagy. In this regard, ELISA kits were provided to measure the levels of RIPK1 (#catalog number: OKCD00437, Aviva Systems Biology, San Diego, California, USA), RIPK3 (#catalog number: MBS1600738, MyBioSource, USA), and MLKL (#catalog number: MBS4501052, MyBioSource, USA), and the analyses were performed based on the manufacturer's protocols. Moreover, ELISA kits were purchased from MyBioSource to measure the levels of LC3 (#catalog number: MBS1600540, MyBioSource, USA), ATG5 (#catalog number: MBS7236767, MyBioSource, USA), and BECN1 (#catalog number: OKEH03267, Aviva Systems Biology, San Diego, California, USA).

### 2.9 The Analysis of Inflammatory Markers

For the measurement of inflammation state in the hepatic tissue, the levels of proinflammatory (TNF‐α and IL‐6) and anti‐inflammatory (IL‐4 and IL‐10) markers were determined using ELISA (MyBioSource) according to the manufacturer's protocols.

### 2.10 The Determination of Stress State

To assess the extent of oxidative stress in hepatic tissues homogenates, the levels of GPx (glutathione peroxidase), MDA (malondialdehyde), GR (glutathione reductase), CAT (catalase), and SOD (superoxide dismutase) enzymes were measured using ELISA kits supplied by MyBioSource, strictly following the manufacturer's protocols.

### 2.11 Statistical Analysis

The obtained data are presented as the mean ± standard deviation (SD) and the difference between studied groups was determined using one‐way ANOVA followed by the Tukey posthoc test after normality analysis using the Kolmogorov–Smirnov test. In this regard, SPSS version 24.0 (IBM, Chicago, IL, USA) was used for statistical analysis, and GraphPad Prism version 8 (San Diego, CA, USA) was used to draw graphics. A *p*‐value < 0.05 was described as significantly different.

## 3 Results

### 3.1 EA Improved the Physical and Biochemical Indices of Rats With NAFLD

**FIGURE 1:**
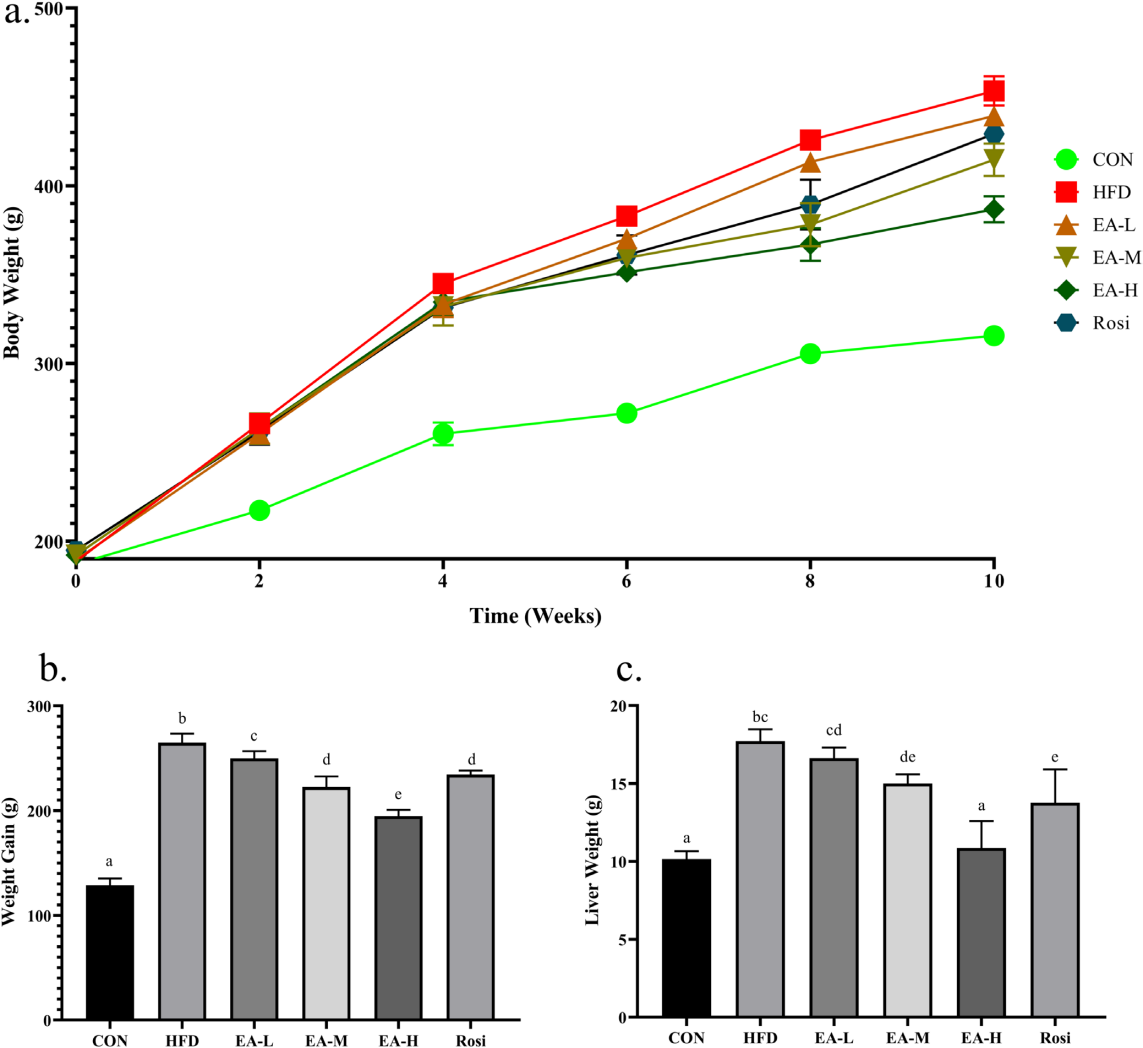
Physical analysis. Total body weight (a), weight gain (b), and the weight of liver tissue (c) in animals with NAFLD were significantly higher than controls. However, the administration of a high dose of EA significantly restored the physical parameters of rats with NAFLD. The statistical analysis results are shown by lowercase letters on the bars, where different letters stand for significant differences between groups (a: Significant difference with b, c, d, e. b: Significant difference with c, d, e. c: Significant difference with d, e. d: Significant difference with e. e: Significant difference with f. bc: Significant difference with a, de, e. cd: Significant difference with a, e. de: Significant difference with bc, a.); a *p*‐value < 0.05 was described as significant.

The present study established a model of HFD‐induced NAFLD in rats using previous research (Qin et al. 2018) to investigate the ameliorative effects of EA administration on disease. The findings of the present study showed that during the treatment there was a significant difference between the studied groups in terms of total body weight (*p*‐value < 0.001, Figure 1). Moreover, in terms of weight gain, groups HFD, EA‐L, EA‐M, EA‐H, and Rosi caused a significant increase of 2.06 times, 1.94 times, 1.73 times, 1.51 times, and 1.81 times, respectively, compared to the CON (*p*‐value < 0.001). However, weight gain in EA‐L, EA‐M, EA‐H, and Rosi groups was significantly lower than that of HFD rats (*p*‐value < 0.001). Furthermore, the weight of the liver tissue in the HFD rats increased 1.75 times in comparison with the CON (*p*‐value < 0.001). There was no significant difference between groups EA‐L and HFD in terms of liver tissue weight (*p*‐value > 0.05), but EA‐M, EA‐H, and Rosi groups caused a significant decrease when compared to HFD animals. Nevertheless, the weight of liver tissue in only EA‐H animals was not different in comparison with the CON group (*p*‐value > 0.05).

**FIGURE 2:**
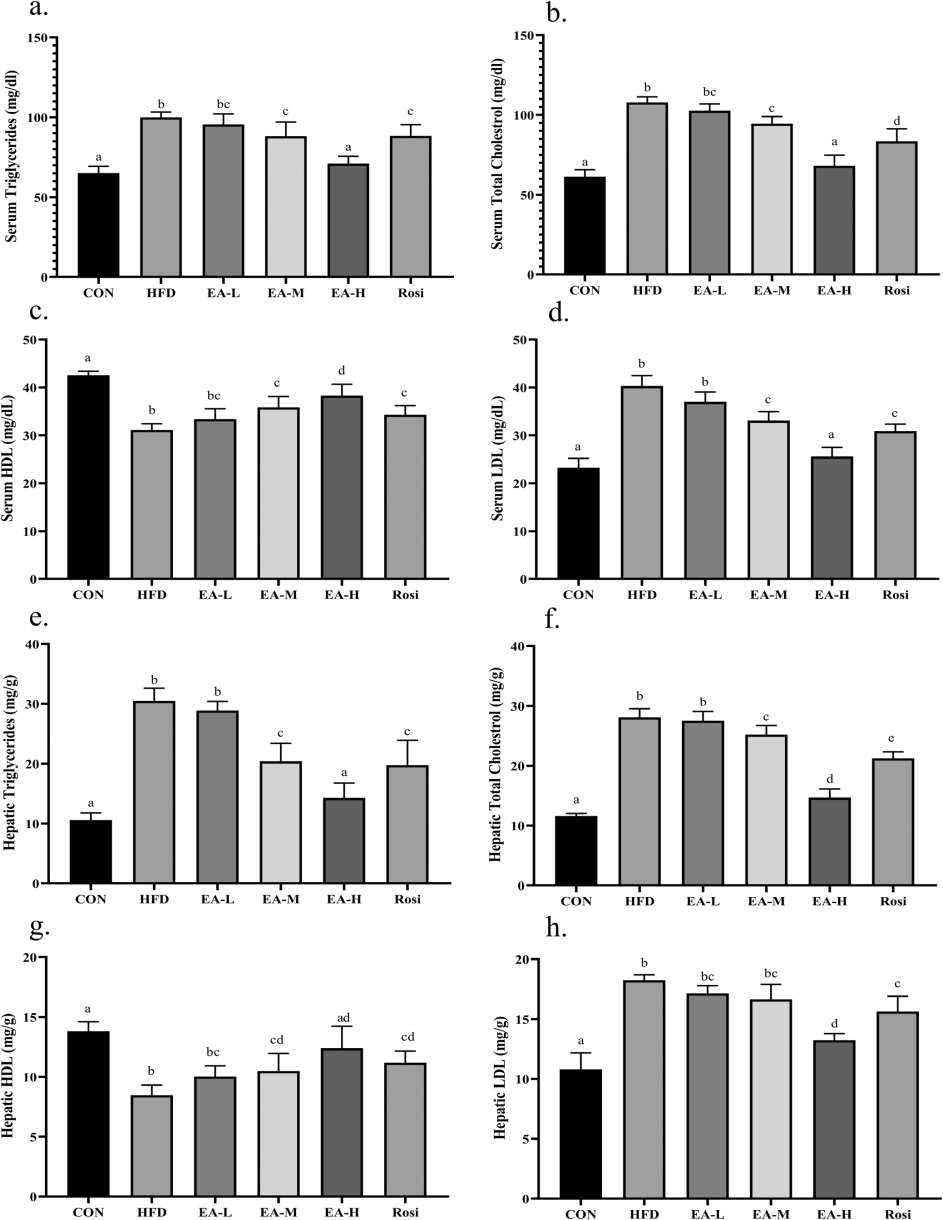
Lipid profile analysis in the serum and hepatic tissue. The levels of TG (a and e, respectively), TC (b and f, respectively), HDL (c and g, respectively), and LDL (d and h, respectively) in the serum and hepatic tissue of NAFLD animals were ameliorated after the administration of EA. The statistical analysis results are shown by lowercase letters on the bars, where different letters stand for significant differences between groups, where different letters indicate significant differences between groups (a: Significant difference with b, bc, c, d, e, cd. b: Significant difference with c, d, e, ad, cd. c: Significant difference with d, e. d: Significant difference with bc, e. e: Significant difference with bc, ad, cd, bc: Significant difference with a, d. cd: Significant difference with a, b, ad: Significant difference with b, c); a *p*‐value < 0.05 was described as significant.

Additionally, the present study investigated the lipid profile in both the sera and hepatic tissues (Figure 2). According to the obtained data, the serum and tissue levels of TG, TC, and LDL in the HFD animals increased significantly in comparison with CON (*p*‐value < 0.001), whereas the level of HDL decreased significantly (*p*‐value < 0.001). Interestingly, administration of the EA at a high dose was able to significantly prevent lipid accumulation in the serum and liver (*p*‐value < 0.05), although the lipid profile in EA‐M and Rosi groups were remarkably different from both the HFD group and CON group (*p*‐value < 0.05).

### 3.2 The Liver Histoarchitecture of Animals With NAFLD Was Improved After Treatment With EA

**FIGURE 3:**
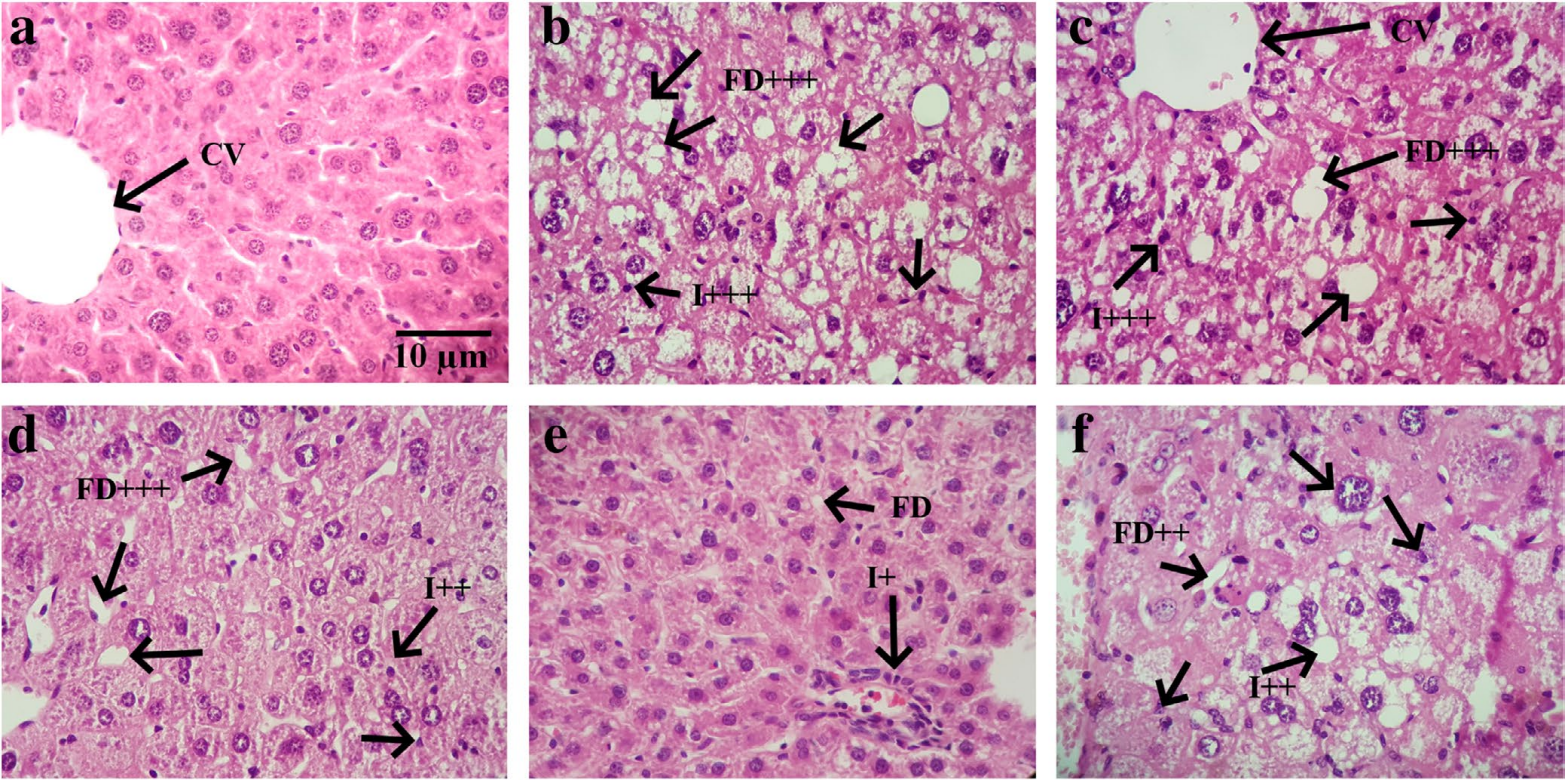
The histological analysis of liver tissue. The findings showed that in control (a), intact hepatic lobular architecture with normal morphology existed. The liver of HFD animals (b) represented marked micro and macro steatotic alterations and moderate interlobular inflammation. Although the administration of low (c) and medium (d) doses of EA and the administration of Rosi (f) had a slight improvement effect on the histological characteristics of liver specimens, high doses of EA (e) had appropriately preserved the morphological appearance of the liver tissue. CV: Central vein; I: Inflammation; FD: Fatty degeneration due to steatosis and ballooning of hepatocytes; +: Mild; ++: Moderate; +++: Severe.

Liver sections of CON animals demonstrated preserved lobular architecture with no presence of significant hepatocytes, ballooning inflammatory infiltration, and fat accumulation (Figure 3). However, HFD animals represented typical micro‐ or macrovesicular steatosis, hepatocytes with vacuolation, lobular infiltration by mononuclear cells and lymphocytes, and abundant fat deposition, fat droplet accumulation, spotty and focal necrosis (Table 2). Interestingly, the high doses of EA administration preserved the liver histology of NAFLD animals.

**TABLE 2:** Histological characteristics of the liver tissue.

Groups	Hepatic architecture		Steatosis (%)	Steatosis type		Inflammation	Hepatocyte vacuolation	Fibrosis	
	Preserved	Lost		Micro‐vesicular	Macro‐vesicular			None	Present
CON	6/6	0/6	10.3 ± 1.8	2/6	4/6	6.7 ± 1.8	6.14 ± 0.94	6/6	0/6
HFD	2/6	4/6	60.5 ± 9.73^a^	5/6	1/6	13.1 ± 4.5^a^	39.75 ± 4.26^a^	0/6	6/6
EA‐L	3/6	3/6	54.3 ± 7.4^a^	5/6	1/6	12.6 ± 2.3^a^	33.48 ± 1.71^a^	1/6	5/6
EA‐M	4/6	2/6	48.2 ± 5.2^a^	3/6	3/6	9.3 ± 3.1^b^	19.05 ± 3.61^b^	2/6	4/6
EA‐H	5/6	1/6	28.9 ± 4.6^c^	2/6	4/6	6.7 ± 2.5^c^	8.75 ± 1.98^c^	5/6	1/6
Rosi	3/6	3/6	43.4 ± 7.8^b^	4/6	2/6	9.5 ± 4.1^b^	22.48 ± 2.14^b^	3/6	3/6

*Note:* Values are presented as number, percent, and mean ± SD.

^a^ Significant difference with only CON group.

^b^ Significant difference with both CON and HFD groups.

^c^ Significant difference with only HFD group, *p*‐value < 0.05 was considered significant.

### 3.3 EA Suppressed Necroptosis in the Hepatic Tissue of Rats With NAFLD

**FIGURE 4:**
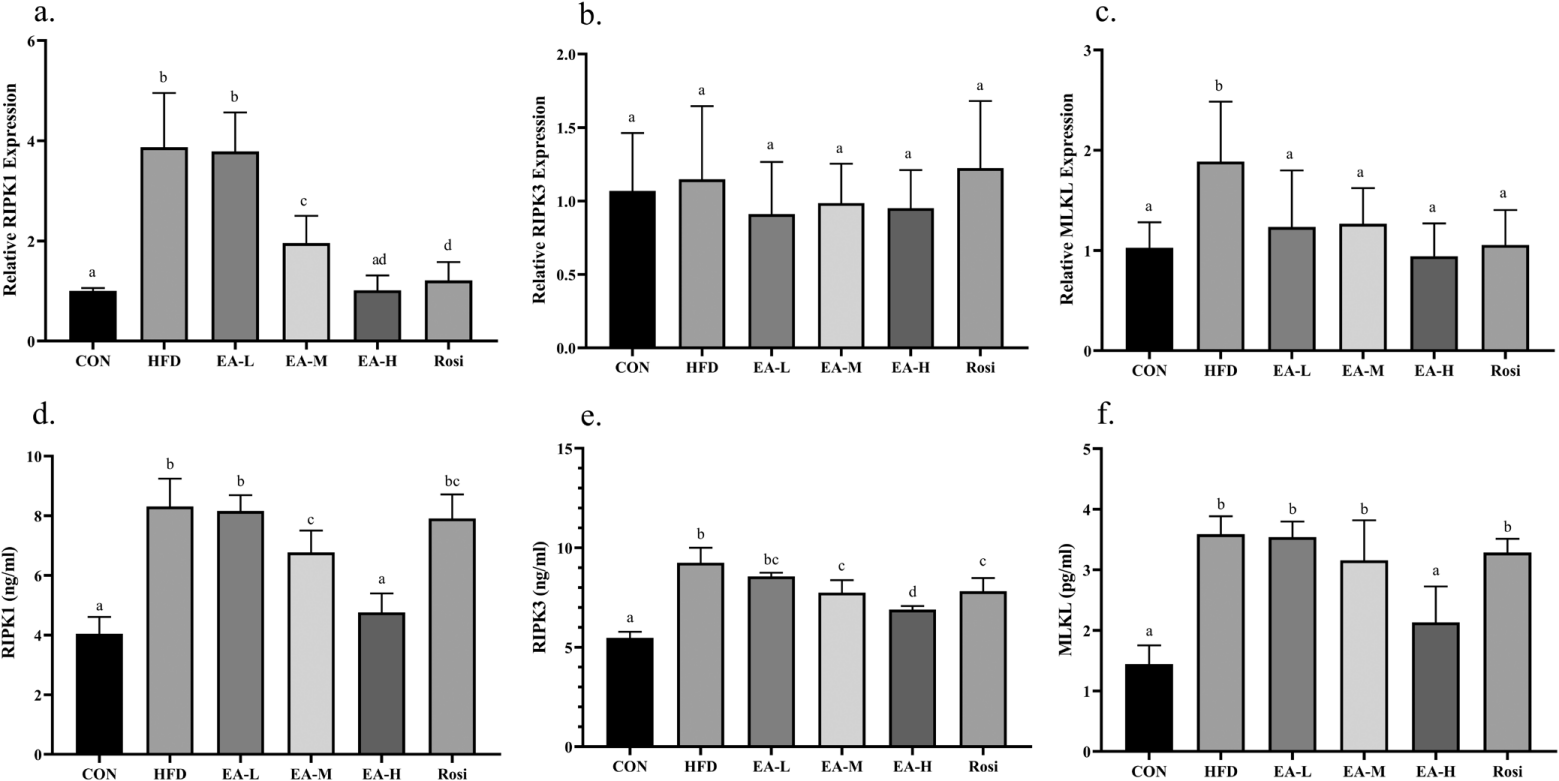
EA suppressed necroptosis in hepatic tissue. The gene expression and protein levels of RIPK1 (a and d, respectively), RIPK3 (b and e, respectively), and MLKL (c and f, respectively) were measured. The statistical analysis results are shown by lowercase letters on the bars, where different letters stand for significant differences between groups, where different letters indicate significant differences between groups (a: Significant difference with b, c, d, bc. b: Significant difference with c, d, ad. c: Significant difference with ad. d: Significant difference with bc. ad: Significant difference with b, c. bc: Significant difference with a, d); a *p*‐value < 0.05 was described as significant.

Liver necroptosis status was investigated to determine the level of three main markers involved in the process of necroptotic death, including RIPK1, RIPK3, and MLKL (Figure 4). Although the expression of *RIPK3* encoding gene did not show a considerable difference when all groups were analyzed (*p*‐value = 0.353), the expression of genes encoding *RIPK1* and *MLKL* in the HFD group had a significant increase of 3.86 times and 1.84 times, respectively, in comparison with the CON (*p*‐value < 0.001). Interestingly, the administration of all the studied doses of EA and the treatment with Rosi caused a significant decrease in *MLKL* gene expression (*p*‐value < 0.05), however, the expression of *RIPK1* was restored to CON levels only when high doses of EA were administrated (*p*‐value > 0.05 in comparison with CON). Also, in HFD, EA‐L, EA‐M, and Rosi groups the levels of proteins RIPK1 (105.79%, 102.05%, 67.72%, and 95.79%, respectively) and MLKL (149.10%, 145.90%, 119.24%, and 128.13%, respectively) proteins were significantly increased compared to the CON (*p*‐value < 0.001), whereas the EA‐H group showed no significant difference neither in terms of RIPK1 level nor in terms of MLKL level when compared to CON (*p*‐value > 0.05). This is even though in all the studied groups, compared to the CON, the level of RIPK3 protein increased significantly (*p*‐vlaue < 0.05), although groups EA‐M, EA‐H, and Rosi had a noticeable decrease in comparison with HFD animals (*p*‐value < 0.001).

### 3.4 EA Promoted Autophagy in Hepatic Tissue of NAFLD Animals

**FIGURE 5:**
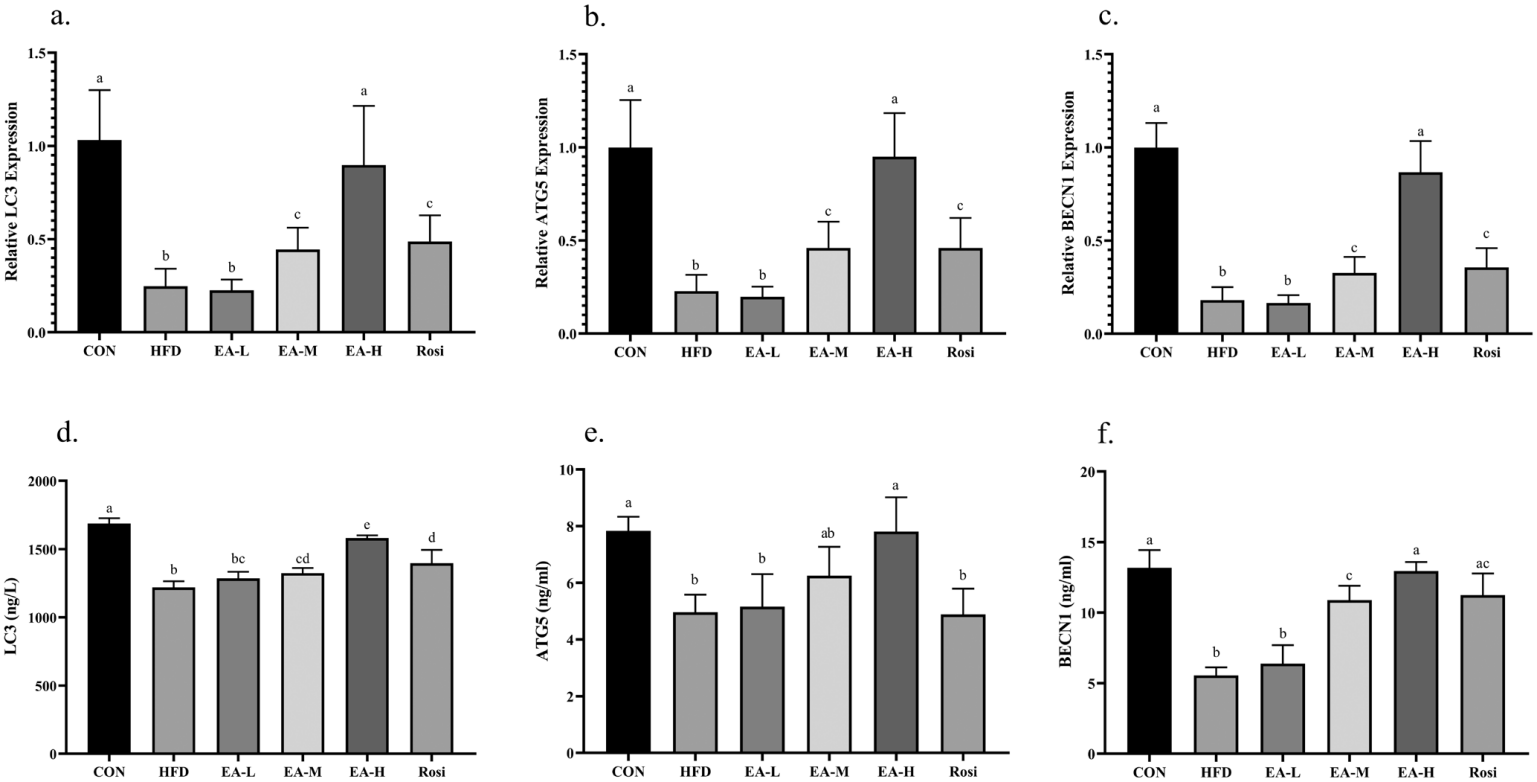
The analysis of autophagic markers in the hepatic tissue. The gene expression and protein levels of LC3 (a and d, respectively), ATG5 (b and e, respectively), and BECN1 (c and f, respectively) were determined using RT‐qPCR and ELISA techniques. The statistical analysis results are shown by lowercase letters on the bars, where different letters stand for significant differences between groups, where different letters indicate significant differences between groups (a: Significant difference with b, c, bc. b: Significant difference with c. c: Significant difference with ab. ab: Significant difference with c, d. bc: Significant difference with a, d); a *p*‐value < 0.05 was described as significant.

The evaluation of the autophagy status in the liver tissue was studied (Figure 5). The obtained data revealed that in the HFD group, the expression of *LC3* and *ATG5* genes had a significant decrease of 56.96% and 77.15%, respectively, in comparison with the CON (*p*‐value < 0.001). Although administration of a low dose of EA did not cause a significant difference in terms of the expression of *LC3* (*p*‐value = 0.999) and *ATG5* (*p*‐value = 0.997) genes compared to the HFD group, the EA‐M and Rosi groups showed a significant difference with both the HFD group and the CON group (*p*‐value < 0.05). Meanwhile, the *LC3, BECN1* and *ATG5* expressions in the EA‐H group had no significant difference from the CON (*p*‐value > 0.05). Similarly, the levels of LC3, BECN1 and ATG5 proteins significantly decreased in the hepatic tissue of rats in HFD, EA‐L, EA‐M, and Rosi groups compared to CON animals (*p*‐value < 0.05), whereas no significant difference between CON and EA‐H animals was obtained regarding the hepatic levels of LC3, BECN1 and ATG5 proteins (*p*‐value > 0.05).

### 3.5 EA Suppressed Inflammation in NAFLD Animals

**FIGURE 6:**
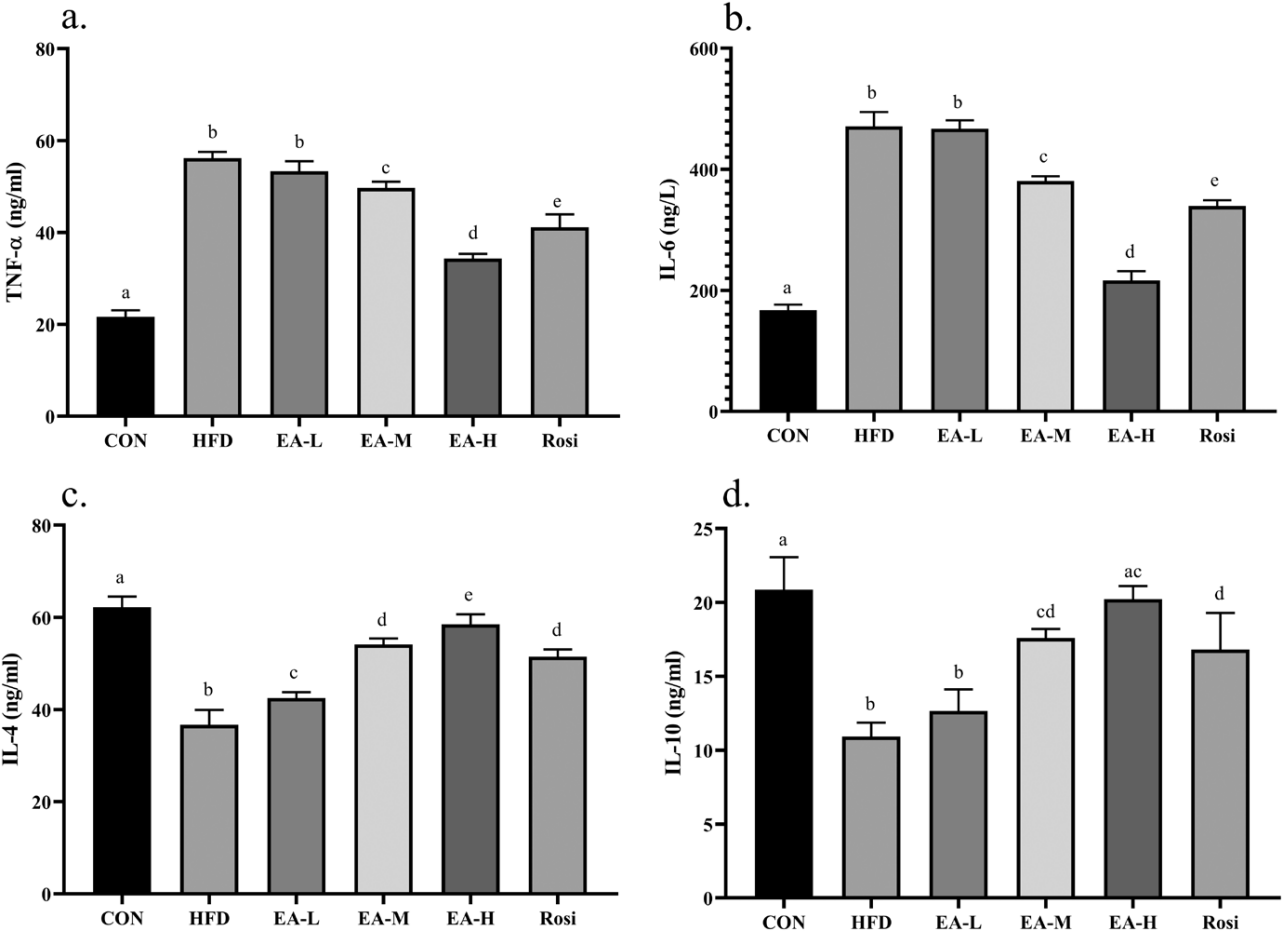
EA inhibited inflammation in NAFLD animals. The findings revealed that EA significantly decreased the levels of proinflammatory cytokines including TNF‐α (a) and IL‐6 (b) while increasing the levels of anti‐inflammatory cytokines, IL‐4 (c) and IL‐10 (d). The statistical analysis results are shown by lowercase letters on the bars, where different letters stand for significant differences between groups, where different letters indicate significant differences between groups (a: Significant difference with b, c, d, e, cd. b: Significant difference with c, d, e, cd, ac. c: Significant difference with d, e. d: Significant difference with e, ac. e: Significant difference with ac, cd. ac: Significant difference with b, d. cd: Significant difference with a, b); a *p*‐value < 0.05 was described as significant.

The state of inflammation in the studied groups was investigated by measuring the serum level of inflammatory markers (Figure 6). The findings showed that HFD caused a significant increase in the levels of TNF‐α (2.59 times) and IL‐6 (2.81 times), whereas the levels of IL‐4 (40.99%) and IL‐10 (47.60%) in the HFD group were significantly lower than the CON (*p*‐value < 0.001). However, medium and high doses of EA as well as administration of Rosi were able to cause a significant difference in terms of the levels of studied inflammatory markers in comparison with HFD‐treated animals (*p*‐value < 0.01).

### 3.6 Oxidative Stress Was Diminished in NAFLD Animals Treated With EA

**FIGURE 7:**
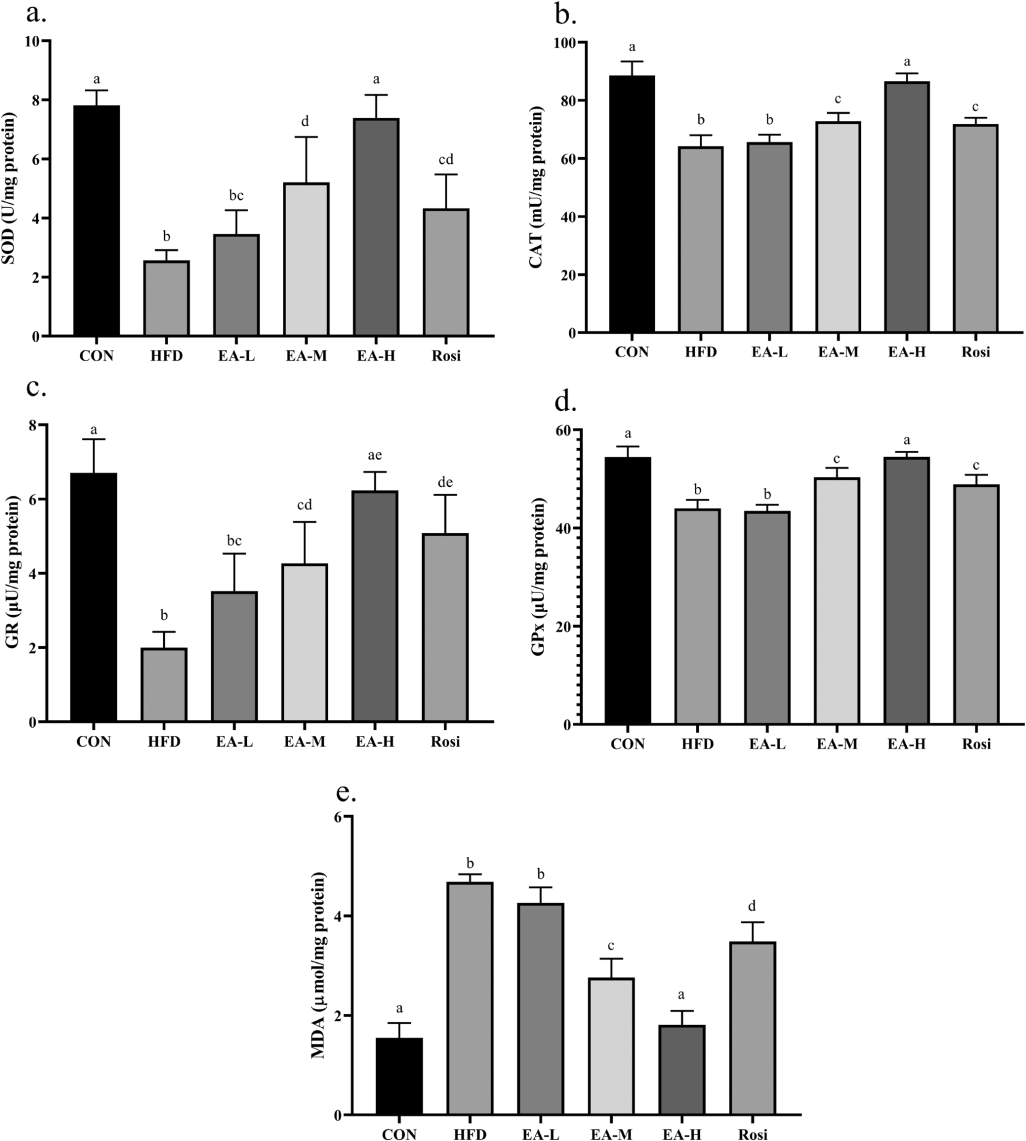
Oxidative stress was diminished in the hepatic tissue of NAFLD animals treated with EA. The levels of SOD (a), CAT (b), GR (c), and GPx (d) were increased after administration of EA, whereas the levels of MDA (e) were decreased. The statistical analysis results are shown by lowercase letters on the bars, where different letters stand for significant differences between groups, where different letters indicate significant differences between groups (a: Significant difference with b, c, d, bc, cd, de. b: Significant difference with c, d, e, cd, ae, de. c: Significant difference with d, ae, de. d: Significant difference with ae, bc. bc: Significant difference with a, d, e. cd: Significant difference with a, b, e. ae: Significant difference with b, c, d. de: Significant difference with a, b, c); a *p*‐value < 0.05 was described as significant.

The present findings showed that in the liver tissue of animals of HFD and EA‐L groups the levels of SOD (67.18% and 55.74%, respectively), CAT (27.54% and 25.96%, respectively), GR (70.19% and 47.55%, respectively), and GPx (19.18% and 20.17%, respectively) significantly declined when a comparison with CON was performed (Figure 7, *p*‐value < 0.01), whereas the level of MDA in HFD and EA‐L groups significantly increased by 3.03 times and 2.75 times compared to the CON, respectively (*p*‐value < 0.001). Although EA‐M and Rosi groups showed significant differences with both HFD and CON groups in terms of the level of markers related to oxidative stress in liver tissue (*p*‐value < 0.05), only the EA‐H group did not show significant differences with CON (*p*‐value > 0.05).

## 4 Discussion

NAFLD is defined as a complex, systemic, and chronic disorder with a multi‐hit pathophysiology. However, there is no definite treatment for NAFLD. Recent findings have shown that cell death mechanisms (Xiao et al. 2023), along with inflammation and stress (Paoli and Cerullo 2023), contribute to the occurrence and progression of the disease. Therefore, the application of herbs with modulating properties of the aforementioned mechanisms has been suggested as a novel strategy for NAFLD management in recent studies. Novelly, the present study aimed to elucidate the effect of EA on an animal model of HFD‐induced NAFLD by investigating the mechanisms involved in programmed cell death including autophagy and necroptosis, as well as analyzing histopathology, metabolic profile, oxidative stress, and inflammation.

The present data revealed that HFD‐induced NAFLD was associated with a significant increase in total body weight, elevated liver weight, and lipid accumulation in serum and hepatic tissue. Moreover, the liver of animals with NAFLD revealed histopathological changes such as steatosis, fibrosis, and hepatocyte vacuolation. However, treatment with a high dose of EA had restorative effects on weight gain and liver weight, prevented lipid accumulation in serum and hepatic tissue, and improved hepatic histomorphology. A variety of studies have associated the NAFLD onset and progression with the disruption of lipid homeostasis, in which dyslipidemia and metabolic syndrome have been described as closely related to the disease (Deprince, Haas, and Staels 2020; Katsiki, Mikhailidis, and Mantzoros 2016). Moreover, lipid excessive accumulation in the form of triglycerides with more than 5% fat in the hepatocytes is considered a main feature of NAFLD patients (Ludwig et al. 1980). Thereby, the attenuation of hyperlipidemia in the serum and liver tissue of animals with NAFLD after the administration of a high dose of EA may suggest this chemical in the management of NAFLD. Indeed, a variety of experimental studies, reviews, and meta‐analyses emphasize the function of phytochemicals in maintaining the homeostasis of glycemic and lipid profiles (Mahdavi et al. 2021; Vinayagam, Xiao, and Xu 2017). In this regard, research has shown that herbs can regulate the production and consumption of carbohydrates and lipids through the modification of upstream signaling pathways (Li et al. 2022; Zhao et al. 2019; Beizavi et al. 2021). Hence, phytochemicals are widely suggested to confront chronic diseases related to metabolism such as type 2 diabetes, polycystic ovary syndrome (Jafari Khorchani et al. 2021), metabolic syndrome, obesity (Poulios et al. 2024), etc.

Programmed cell death mechanisms are intrinsically correlated with inflammatory states of the liver tissue and are considered crucial contributors to governing the clinical outcomes of metabolism‐related liver diseases (Gautheron, Gores, and Rodrigues 2020). It is widely suggested that different forms of programmed cell death pathways including necroptosis and autophagy are related to NAFLD. Hepatocellular death is assumed as a pivotal player in the onset and development of NAFLD, along with inflammatory responses within hepatic tissue, dyslipidemia, and metabolism dysregulation (Aravinthan et al. 2013; Carranza‐Trejo et al. 2021). The involvement of necroptosis in intracellular bioenergetic regulation and inflammation through the contribution of RIPK3 to the necroptotic death of white adipocytes and the hepatocytes mitochondrial bioenergetics highlights this type of cell death as a marker of NAFLD (Islam, Afonso, and Rodrigues 2022; Leven et al. 2021). Additionally, emerging documents support the major participation of defective autophagy in NAFLD pathogenesis since this type of regulated cell death regulates lipid metabolism, modifies insulin resistance, and mediates the survival of hepatocytes. However, in inflammatory states, autophagy initiates the regulated death of hepatocytes (Wu, Zhang, and Chan 2018).

Accordingly, the findings of the present study demonstrated that in the liver tissue of rats with HFD‐induced NAFLD, necroptosis was remarkably enhanced while autophagic flux was suppressed. This is even though administration of a high dose of EA inhibited necroptosis and induced autophagy in animals with NAFLD. Similarly, previous studies have shown that EA is able to prevent liver diseases, including NAFLD, by suppressing cell death and promoting autophagy (Aishwarya, Solaipriya, and Sivaramakrishnan 2021; Elseweidy et al. 2022; Jin et al. 2022). It is widely suggested that necroptotic‐mediated death of liver cells is generally accompanied by inflammation, elevated infiltration of immune cells, and the induction of oxidative stress (Chavoshinezhad et al. 2023; Mohammed et al. 2021). Therefore, the ameliorative properties of EA in animals with HFD‐induced NAFLD may be related to its anti‐inflammatory and anti‐stress properties. The obtained data revealed that inflammation and oxidative stress were considerably present in HFD‐induced NAFLD animals, whereas EA effectively inhibited the inflammation and stress induced by the disease. Concordantly, the feature of phytochemicals, particularly EA, in suppressing inflammation and stress contributes to preserving the survival of cells, whether against environmental factors or genetic disorders (Senavirathna et al. 2024; Zhao et al. 2021). Therefore, one may conclude that EA improves NAFLD in an animal model by suppressing stress and inflammation, inhibiting necroptosis, promoting autophagy, and preventing hepatic lipid accumulation.

Although the development of animal models of NAFLD is an acceptable approach to investigating the disease, the differences from NAFLD in humans are plausible, which can be considered the major limitation of the present study. There is growing evidence elucidating the upstream regulators of programmed cell death in physiological and pathological conditions. Nevertheless, the lack of determining the upstream mechanisms regulating autophagy and necroptosis can be considered another important limitation. Therefore, it is necessary to conduct further animal studies to investigate the effect of EA on the upstream regulators of autophagy and necroptosis (such as NF‐kB, mTOR, AMPK, etc.) and subsequently conduct clinical trials to validate the desired effects of EA on NAFLD.

## 5 Conclusion

The current findings revealed that EA prevented lipid accumulation, inhibited necroptosis, promoted autophagy, and suppressed inflammation and stress in the liver tissue of animals with NAFLD. Thereby, the obtained data may suggest EA as a novel therapeutical strategy for the management of NAFLD. Nevertheless, further studies are pivotally required to elucidate the efficiency and safety of EA in the treatment of NAFLD.

## Author Contributions

**Zhuoheng Li:** conceptualization (equal), supervision (equal), writing – review and editing (equal). **Juan Li:** conceptualization (equal), supervision (equal), writing – review and editing (equal). **Shuli He:** formal analysis (equal), investigation (equal), methodology (equal), writing – original draft (equal), writing – review and editing (equal). **Jun Chen:** formal analysis (equal), investigation (equal), methodology (equal), writing – original draft (equal), writing – review and editing (equal). **Chengjun Deng:** conceptualization (equal), supervision (equal), writing – review and editing (equal). **Jintao Duan:** conceptualization (equal), supervision (equal), writing – review and editing (equal).

## Ethics Statement

The present study was designed and performed based on the international guidelines related to animal care and the National Institute of Health (NIH). The application of animals and procedures of this study were reviewed and approved by the ethics committee of the university and performed according to ARRIVE guidelines.

## Consent

The authors have nothing to report.

## Conflicts of Interest

The authors declare no conflicts of interest.

## Data Availability

The datasets used and/or analyzed during the current study are available from the corresponding author upon reasonable request.
